# Nasal tip projection in facial aesthetics: a study on diversity of preferences in the regions of Brazil

**DOI:** 10.1016/j.bjorl.2026.101905

**Published:** 2026-09-22

**Authors:** Giuliana Beduschi, Michelle Lavinsky, Raphaella de Oliveira Migliavacca, Samuel Afonso de Freitas Toledo, Bárbara Vieira Motta, Sofia Michaelsen Premebida

**Affiliations:** a Hospital de Clínicas de Porto Alegre, Porto Alegre, RS, Brazil; b Hospital Moinhos de Vento, Porto Alegre, RS, Brazil; c Universidade Federal do Rio Grande do Sul, Porto Alegre, RS, Brazil

**Keywords:** Rhinoplasty, Facial aesthetics, Nasal tip projection

## Abstract

**Objectives:**

To provide further insights into the preferences of Brazilians concerning nasal tip projection.

**Methods:**

Observational analytical study. Answers from the general population and plastic surgeons from all regions of Brazil were collected using a form that randomly presented 5 different models of nasal tip projection and asked the informants to select the image that they considered the most beautiful.

**Results:**

Base projection was the most chosen one (48.2%), which corresponds to the Crumley 2 projection. There was no significant difference between the choices of surgeons and those of the general population. The analysis by regions showed a tendency in the southeast region for the informants to choose a less projected nose (projection −1), which corresponds to the Goode projection, unlike the informants in the South and Northeast regions, which opted for the base projection.

**Conclusion:**

The projections most chosen by the population were Crumley 2 and Goode, which correspond to less pronounced projections, unlike the results found in the US. In the southeast region, there was a tendency for a less projected nasal profile in comparison to other regions.

**Level of evidence:**

Level 3

## Highlights

• The study population tend to prefer projections Crumley 2 and Goode.
• Unlike North America, our population tend to prefer less marked projections.
• The southeast region presents a tendency to prefer less projected nasal profiles

## Introduction

Rhinoplasty is one of the most common surgeries in the world.^1^ During the COVID-19 pandemic, people’s noses were visually prominent on both mobile screens and video calls, and on live online broadcasts. As the nose is at the center of the face, it plays an active role on the observer’s aesthetic impression of the latter.^2^ Therefore, rhinoplasty is important because it has the power to modify such a relevant organ of the face.

It is known that for surgical success and consequent patient satisfaction, surgeons must not only master the most appropriate techniques for each case, but also be aware of their patients’ preferences and their own.^3^ Broer et al. (2012) examined nasal shape preferences among more than 1,226 people among plastic surgeons and laymen in 50 countries and found that the general public in Latin America prefers more projected noses compared to the population of respondents in North America.^3^ In 2014, Ahmed and colleagues found that the Crumley 1 nasal projection was more closely correlated with facial attractiveness.^4^ Sun, Sijie et al. (2021) conducted a similar survey with the Chinese population and analyzed 703 answers from plastic surgeons and the general population, and they found no difference in preferences between the regions of China when evaluating the ratio between nasal projection and the length of the nasal dorsum; the most chosen ratio was 0.63.^5^

Because Brazil has continental dimensions and, unlike China, has been colonized by different peoples, Brazilians have diverse phenotypic traits and cultures^6^; therefore, it can be assumed that there are different preferences regarding nasal projection in face aesthetics. A search in the main current databases found no record of a similar study in the Brazilian territory. The present study is the first to address the preferences of Brazilians regarding nasal tip projection in facial aesthetics by analyzing the results found in different regions of the country. This study is expected to help improve medical-patient communication in preoperative surgical planning, and contribute new data to the exchange of experiences in doctor-to-doctor communication in the scientific academic environment. This way, surgeons and patients will hopefully be able to achieve better rhinoplasty results.

## Methods

A form was designed using the Google Forms platform. After giving their informed consent, the respondents were directed to the next page, which contained general questions, including demographic data (age, gender, profession, state of Brazil, ethnicity, personal history of plastic surgery). After that, the respondents were shown profile photos of the same person, which had been edited with 5 different nasal projections ‒ using the Air Brush (© 2022 Pixocial, Inc.) application ‒ with a straight nasal dorsum (named projection −2, −1, base, +1and +2), arranged in random order. The respondents were asked to choose the image that they considered the most beautiful (Fig. 1, Fig. 2). The form was configured in a way that each person answered the questionnaire only once.

**Fig. 1:**
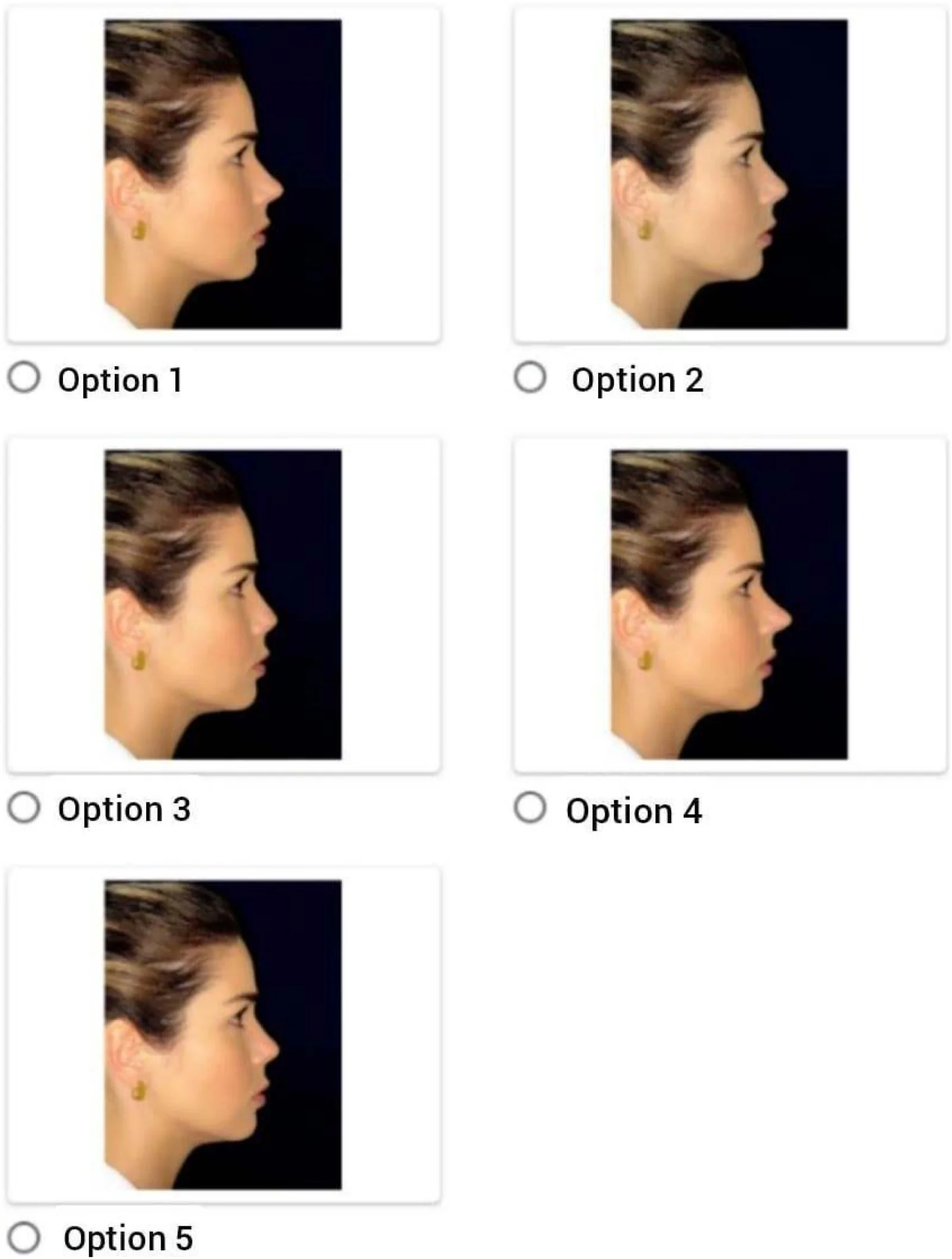
Which of the profiles below do you think is the most beautiful?

**Fig. 2:**
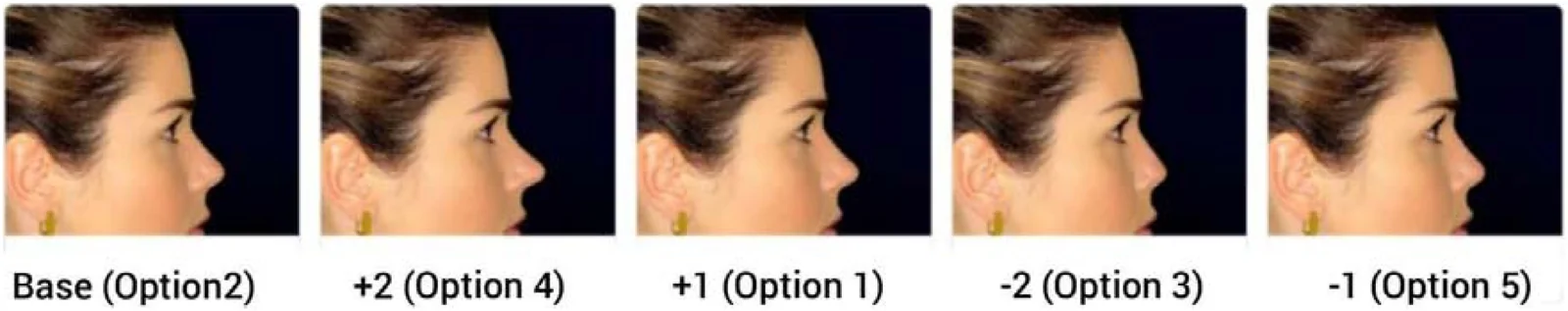
Key to the projections and the options.

The link was initially sent to members of the Brazilian Academy of Facial Plastic Surgery, and to e-mail groups of students and staff of the Hospital de Clinicas de Porto Alegrel (HCPA). It was also shared in WhatsApp groups which included people from different Brazilian states and also via Instagram. During the research study, to collect enough answers from all regions of Brazil, people from the states of the missing regions were actively contacted and sent the link of the survey and asked to share it with their network of friends and local acquaintances. Only Brazilian participants, over 18-years old, were included.

Data was collected only from the form filled in by the informants, and it was organized in a separate Google spreadsheet for subsequent statistical analysis. No secondary data sources were used. The information was stored on personal computers and on Google Drive on an institutional email account of the HCPA. This data was treated anonymously, according to the Brazilian General Data Protection Law. Statistical analysis was performed together with the Statistics Department of the HCPA, and Chi-square tests were performed.

## Results

A total of 488 people from 21 Brazilian states answered the survey. Among all informants, 370 (75.8%) self-reported as women, 117 (24.2%) as men, and 1 person self-reported as non-binary. Of the 39 facial plastic surgeons who participated in this study (plastic surgeons or otorhinolaryngologists), 28 were women and 11, men. The respondents’ age ranged from 18- to 76-years with an average age of 35.05-years. Regarding the choice of profiles with different nasal projections, 48.2% of the respondents selected the base profile; 33.6% chose projection −1; and 12.5% chose projection +1. Projection −2 was chosen by 5.1% of the participants and projection +2 only by 0.6% of the informants (Fig. 3).

**Fig. 3:**
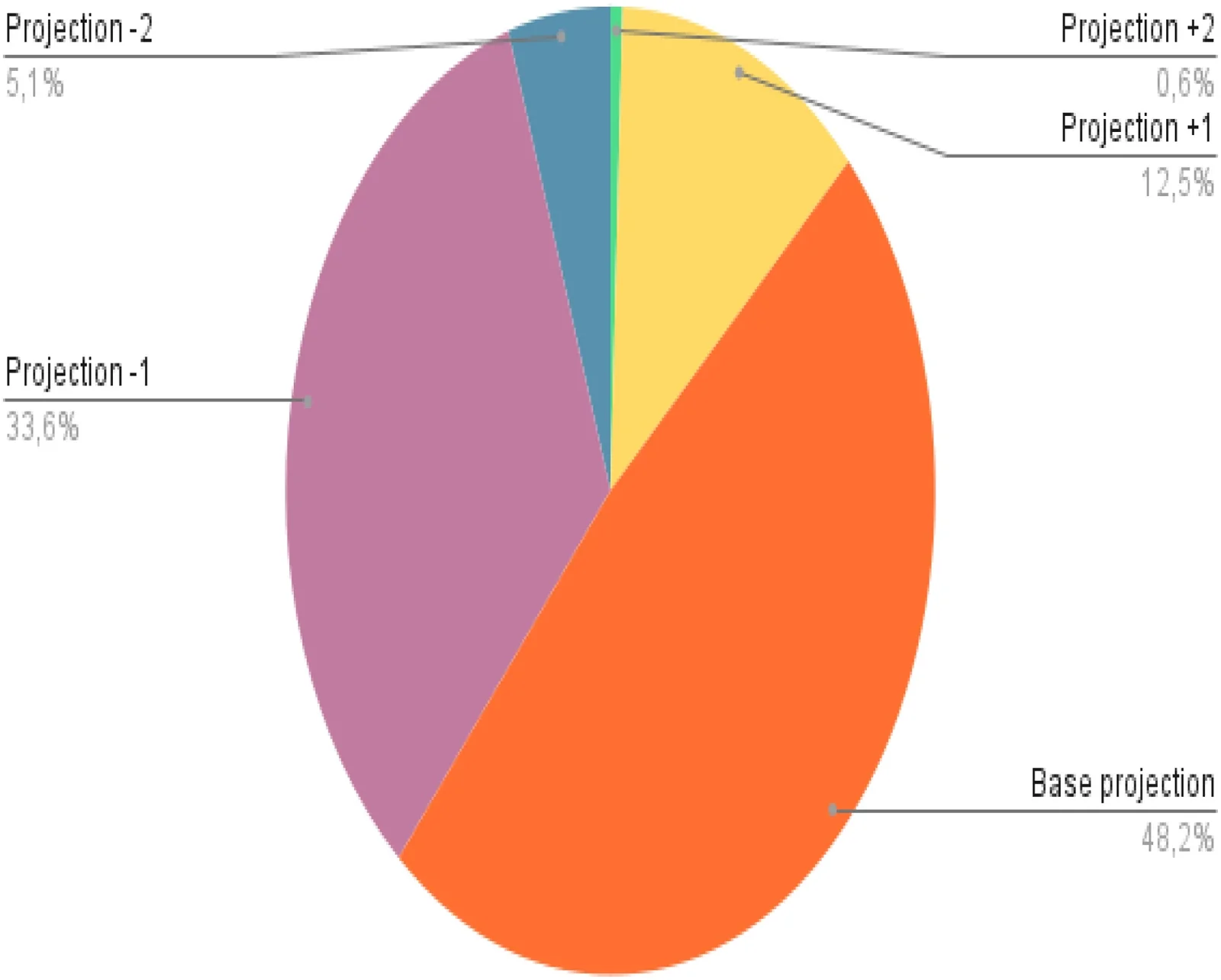
Which of the profiles below do you think is the most beautiful?

Most of the informants were moderately interested in aesthetics (44.1%) while 37.5% of the study population was very and extremely interested in aesthetics (Fig. 4).

**Fig. 4:**
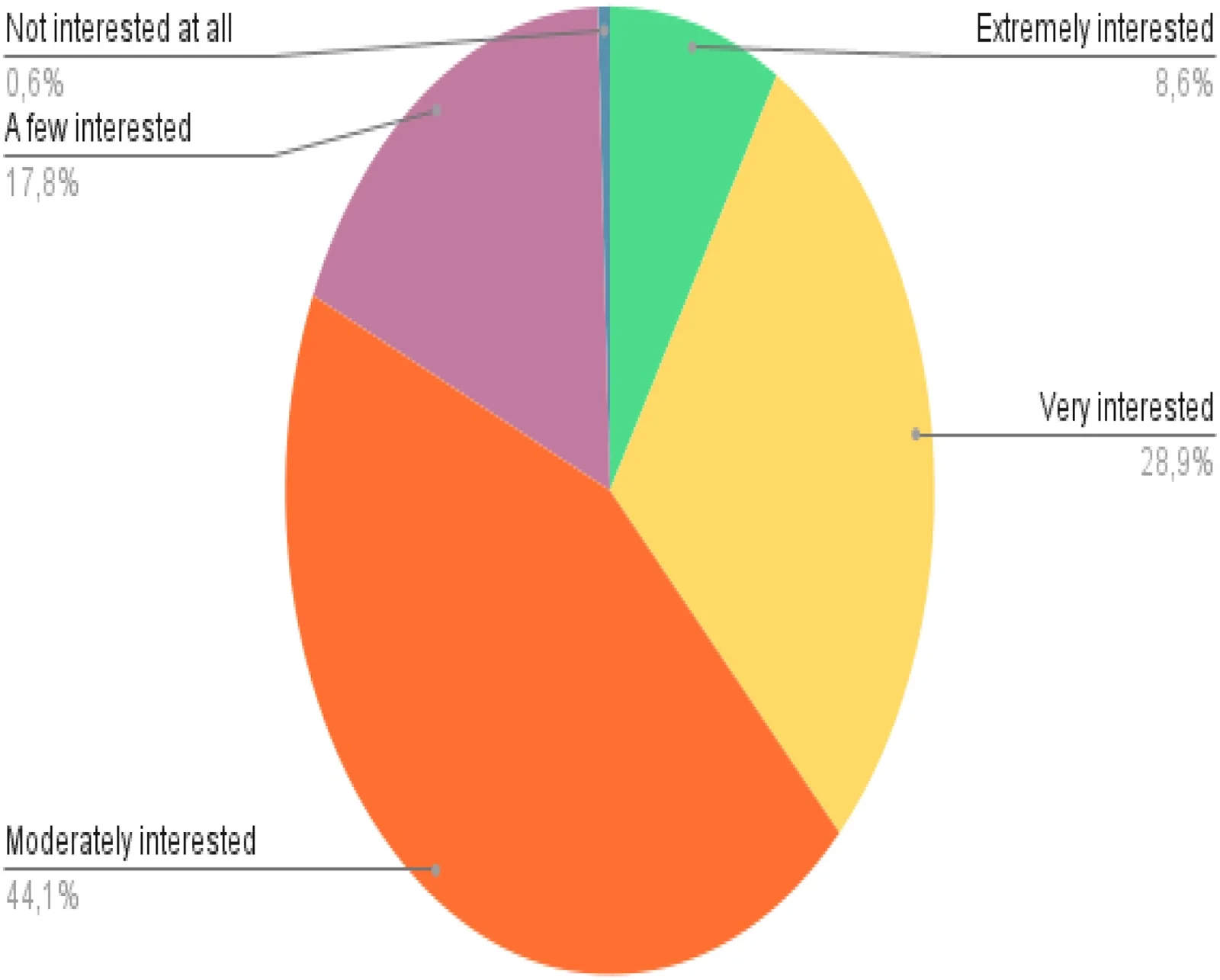
Do you think you are interested in aesthetics?

The analysis of interest in aesthetics, according to the chosen projection, showed no statistical significance (Pearson’s Chi-Square test: p = 0.555) (Table 1).

**Table 1:** Analysis of interest in aesthetics.

	Projection −2	Projection −1	Base Projection	Projection +1
Hardly interested	4.7% (4)	32.6% (28)	47.7% (41)	15.1% (13)
Moderately interested	4.2% (9)	34.6% (74)	50.5% (108)	10.7% (23)
Very interested	5.0% (7)	35.7% (50)	47.1% (66)	12.1% (17)
Extremely interested	11.9% (5)	28.6% (12)	40.5% (17)	19% (8)

As for schooling, 86.03% of the study population had earned an undergraduate or a graduate degree. The plastic surgeons’ choices of profiles did not differ statistically from those of the general population, including their percentages (Fisher's Exact test p = 0.954) (Table 2).

**Table 2:** Plastic surgeons’ choices.

	Projection −2	Projection −1	Base Projection	Projection +1	Projection +2
Plastic Surgeon (39)	5.1% (2)	30.8% (12)	53.8% (21)	10.3% (4)	0
Other (449)	5.1% (23)	33.91% (152)	47.7% (214)	12.7% (57)	0.7% (3)

The analysis of answers according to the regions of Brazil, showed that 74.6% of the informants were from the South, 8.6% from the Southeast, 1.6% from the Midwest, 14.1% from the Northeast, and 1% from the North. Owing to the small number of answers from the North and the Central-West, these regions were excluded at the time of analysis not to overly distort the results.

There was no significant difference in the preferences between the regions, but although most of the respondents prefer the base projection profile, the informants in the Southeast region tend to prefer the profile with projection −1 (Pearson's Chi-Square test: p = 0.525) (Table 3)

**Table 3:** Preferences between the regions.

	Projection −2	Projection −1	Base Projection	Projection +1	Projection +2
South (364)	4.7% (17)	33.5% (122)	49.4% (180)	11.6% (42)	0,8% (3)
Southeast (42)	9.5% (4)	40.5% (17)	33.3% (14)	16,7% (7)	0% (0)
Northeast (69)	5,8% (4)	34,8% (24)	46,4% (32)	5,8% (4)	7,2% (5)

## Discussion

To our knowledge, this is the first study with Brazilians that addresses preferences regarding ideal nasal projection on a female face. Profile photos were edited in a specific application to produce 5 profiles in different nasal projections, which were randomly presented to informants that were asked to choose the one that they considered the most beautiful. In this study, nasal projection is the posterior-to-anterior distance that the nasal tip extends in front of the facial plane.^7^

It was found that the preferences of the general population are in line with the those of facial plastic surgeons, as reported by Sun^5^ but unlike the findings of Broer’s work.^3^

The study by Devcic et al.,^8^ which correlated facial attractiveness with the main quantitative methods of ideal nasal projection reported in the literature (Baum, Powell, Simons, Goode, Crumley 1, and Crumley 2), reported that the projections of Goode and Crumley 1 and Crumley 2 had a greater impact on aesthetic facial perception.

According to Goode, the ideal nasal projection uses a triangle where one side passes through the vertex of the nasofrontal angle through the alar fold; the second side is a perpendicular line passing through the tip-defining point, and the third side is a line drawn to complete the rectangular triangle using the nasal dorsum; the ratio between dorsum length and the distance from the alar fold to the tip-defining point should range between 0.55 and 0.6.^9^ Crumley’s projection 1 is the ratio between the length of the vermilion border of the upper lip to the vertex of the nasofrontal angle, and a second line perpendicular to that angle, through the tip-defining point of the nose. Ideally it is 0.283. Crumley also presented a second ideal projection (Crumley 2) that uses Goode's triangle, but extends the posterior line through the mandibular profile; the ratio of this line with the perpendicular line passing through the tip-defining point, should ideally be 0.2364.^7^

Thus, the projection −2 corresponds to Goode’s formula (2.2/3.8 = 0.57). We also consider that the projection −1 corresponds to that of Goode’s, according to the following calculation: 2.4/3.9 = 0.61. The projection +1 corresponds to Crumley 1 (1.8/6.2 = 0.29) and the base projection to Crumley 2 (2.6/11 = 0.236). The measurements were performed as shown Fig. 5.

**Fig. 5:**
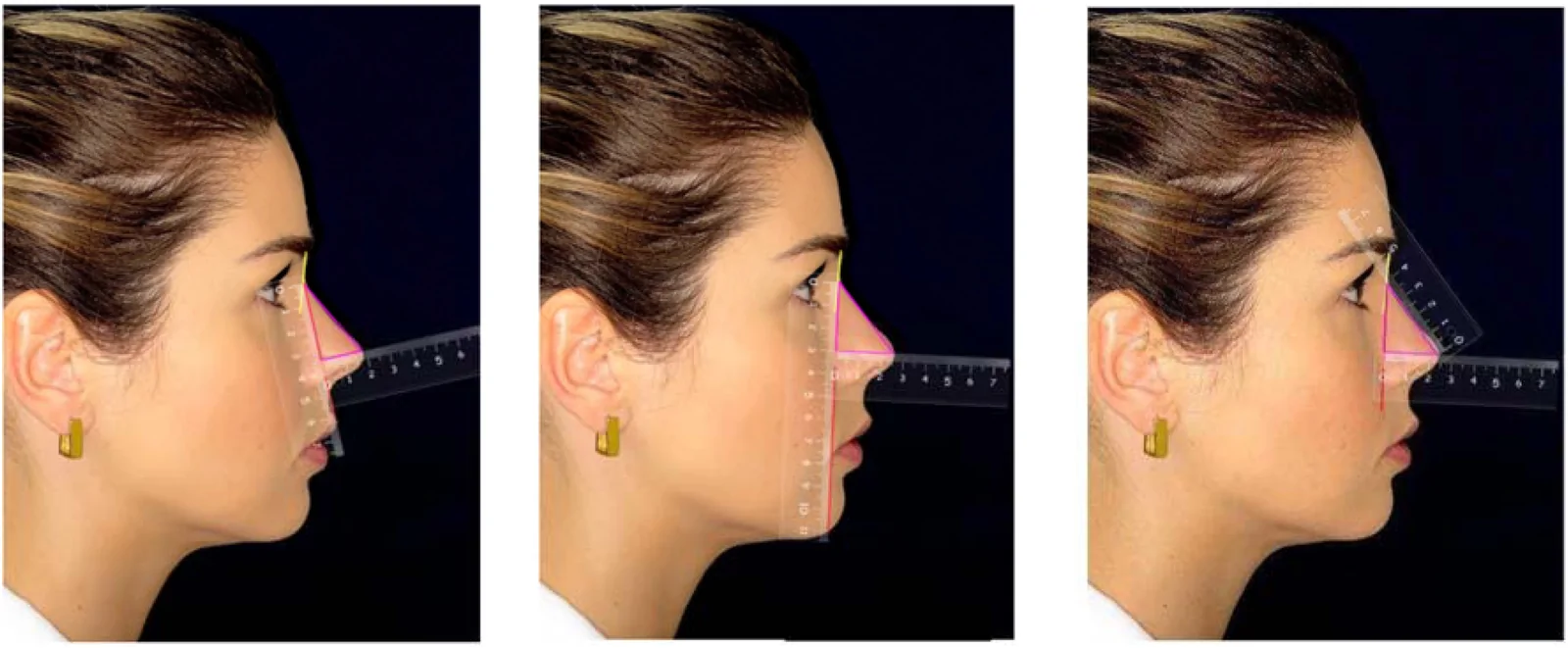
(A) Crumley 1 - Projection +1. (B) Crumley 2 - Base Projection. (C) Goode - Projection −1.

In our study, the profile most chosen by the study population (48.2%) was the “base profile” which corresponds to the ideal projection of Crumley 2; secondly, 33.6% of the respondents chose the “projection −1” which corresponds more approximately to Goode’s projection; 12.5% of the respondents chose the “projection +1”, which corresponds to Crumley 1. The projection −2, which also corresponds to Goode’s projection, was chosen by only 5.1% of the informants. Interestingly, this result differs from the findings by Ahmed et al.,^4^ who evaluated ideal nasal projection preferences and nasolabial angle among 3978 women aged 18- to 25-years old who detected that the Crumley 1 projection was the most attractive among the same methods analyzed in the present study (Goode, Crumley 1, and Crumley 2). Our finding also differs from those in Broer’s work,^3^ which reported that respondents from Latin America prefer more projected noses compared to those from North America while our results show a preference for less prominent noses among the 5 proposed projections. Also, our work has similar results to those found in the study of Sun et al.,^5^ whose most chosen nasal projection (NDL/NTP) was 0.63, that is, between the projection −1 and the base projection in our study - precisely the most chosen ones among our study population.

In addition, our study differed from the study by Broer^3^ regarding the preference of plastic surgeons and the general population. In that study, it was found that surgeons preferred more projected noses than the general population, while in our work and in the study of Sun et al.,^5^ there is no significant difference between the nasal projection choices made by surgeons and by laypersons.

There is no statistically significant difference when preferences were analyzed according to the region of Brazil, even though the North and Central-West regions were excluded and there were very few choices of projection +2. However, there is a certain tendency for the informants in the Southeast region to choose the least projected noses. However, we could not determine whether this result is due to size of the southeast sample or other unidentified factors. According to our study, there is no statistically significant relationship between interest in aesthetics and choice of profiles with more or less projected noses.

This study was performed with a convenience sample, which can be considered a limitation, formed by a group with a majority of females, with a high level of education and high purchasing power. In addition, the measurement inaccuracies inherent to the method of choice can also be considered as limitations. The external validity of the work is impaired because most respondents are from the southern region of Brazil.

Furthermore, it is important to highlight that the Brazilian population is highly mixed, exhibiting phenotypic characteristics from multiple ethnic backgrounds. Consequently, establishing a single aesthetic standard for Brazilians is particularly challenging, as considerable variation exists in nasal morphology, as well as in other facial features that may influence aesthetic preferences.^8^

In the present study, the photographs used as models depicted a caucasian leporine nasal profile. Although the study was designed exclusively to evaluate nasal projection, we acknowledge that participants whose nasal characteristics differed from those of the selected model may have chosen the option that best aligned with the model’s characteristics. Therefore, such selections may not necessarily reflect their true aesthetic preference regarding nasal projection for other nasal profiles.

Nevertheless, distinguishing this effect would be difficult given the wide range of phenotypic profiles within the Brazilian population. Addressing this limitation would require the inclusion of multiple representative models and, ideally, in-person assessments to determine which model best corresponded to each participant. Such methodological adjustments, however, would render a nationwide study impractical without collaboration from research centers across different states.

## Conclusion

This is the first study conducted with the Brazilian population that addresses preferences regarding ideal nasal projection on a female face. The study population tend to prefer Crumley 2 and Goode, which correspond to less marked projections, unlike the findings reported in studies performed in North America. The southeast region presents a tendency to prefer less projected nasal profiles than the other Brazilian region.

## ORCID ID

Giuliana Beduschi: 0009-0006-2949-5838

Michelle Lavinsky: 0000-0001-7791-0019

Raphaella de Oliveira Migliavacca: 0000-0002-5099-7115

Samuel Afonso de Freitas Toledo: 0009-0000-6185-397X

## Funding

No funding.

## Data availability statement

The authors declare that all data are available in repository.

## Declaration of competing interest

The authors declare no conflicts of interest.
